# The RTR complex as caretaker of genome stability and its unique meiotic function in plants

**DOI:** 10.3389/fpls.2014.00033

**Published:** 2014-02-12

**Authors:** Alexander Knoll, Susan Schröpfer, Holger Puchta

**Affiliations:** Botanical Institute II, Karlsruhe Institute of TechnologyKarlsruhe, Germany

**Keywords:** RTR complex, RecQ helicases, topoisomerase 3 alpha, RMI1, DNA repair, homologs recombination, meiotic recombination, gene conversion

## Abstract

The RTR complex consisting of a RecQ helicase, a type IA topoisomerase and the structural protein RMI1 is involved in the processing of DNA recombination intermediates in all eukaryotes. In *Arabidopsis thaliana* the complex partners RECQ4A, topoisomerase 3α and RMI1 have been shown to be involved in DNA repair and in the suppression of homologous recombination in somatic cells. Interestingly, mutants of At*TOP3A* and At*RMI1* are also sterile due to extensive chromosome breakage in meiosis I, a phenotype that seems to be specific for plants. Although both proteins are essential for meiotic recombination it is still elusive on what kind of intermediates they are acting on. Recent data indicate that the pattern of non-crossover (NCO)-associated meiotic gene conversion (GC) differs between plants and other eukaryotes, as less NCOs in comparison to crossovers (CO) could be detected in *Arabidopsis*. This indicates that NCOs happen either more rarely in plants or that the conversion tract length is significantly shorter than in other organisms. As the TOP3α/RMI1-mediated dissolution of recombination intermediates results exclusively in NCOs, we suggest that the peculiar GC pattern found in plants is connected to the unique role, members of the RTR complex play in plant meiosis.

## INTRODUCTION

**FIGURE 1:**
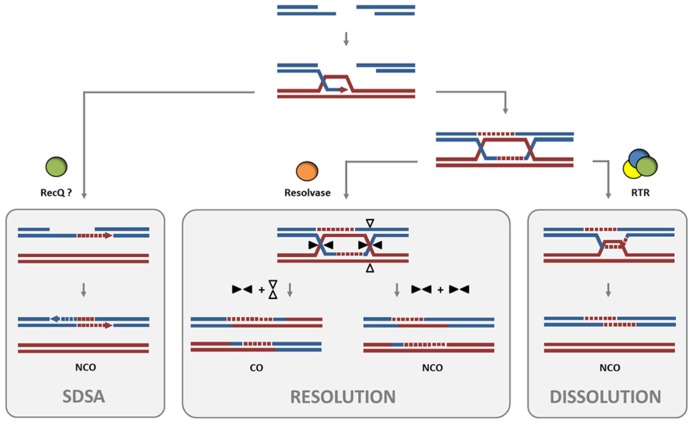
**Dissolution of dHJs by the RTR complex in comparison to resolution.** After the formation of a DSB and resection of the break ends, strand invasion into a double-stranded donor molecule forms a D-loop intermediate. Rejection of the invaded strand after its elongation and subsequent annealing to the second free break end enables DSB repair by SDSA without formation of COs. RECQ4A and its homolog in yeast, Sgs1, have been found to promote this SDSA pathway. Following the formation of a dHJ intermediate from the D-loop during HR, two pathways have been described to separate the two linked dsDNA molecules. In the dissolution pathway promoted by the RTR complex, a RecQ family DNA helicase branch migrates the two junctions to form a hemicatenane structure. This hemicatenane is processed by the type IA topoisomerase into a NCO product. The non-catalytic protein RMI1 interacts with the RecQ helicase and topoisomerase and promotes the dissolution reaction. Alternatively, the dHJ structure can be processed by a structure-specific endonuclease, also called a HJ resolvase, in the resolution pathway. Here, depending on the orientation of the cut axis (arrowheads), either CO or NCO products are possible. Other orientations of the cuts by the resolvase would lead to similar CO or NCO outcomes and are not shown.

The processing of DNA intermediates which occur during homologous recombination (HR) is an indispensable step for the exchange of information between the parental chromosomes in meiotic cells and also ensures the genomic stability in somatic cells. Some pathways of HR are based on the formation of joint molecules like double Holliday Junctions (dHJs), which in eukaryotes can be either resolved by the endonucleolytic action of HJ resolvases such as MUS81 or by dHJ dissolution (**Figure 1**). The dissolution reaction is mediated by the conserved RTR complex, which is named after the interacting complex partners: a RecQ helicase, a type IA topoisomerase, and a structural protein RMI1. With regard to the maintenance of genome stability, the RTR complex plays a crucial role in suppression of crossover (CO) products in somatic cells, because the dissolution of dHJs results exclusively in non-crossover (NCO) products (Wu and Hickson, 2003). In the first step of dHJ dissolution, the branch migration activity of the RecQ helicase is required to push the two HJs toward each other, thereby generating a so-called hemicatenane intermediate. Subsequently, the hemicatenane is processed by the type IA topoisomerase which acts as a single-stranded DNA decatenase (Yang et al., 2010).

The human helicase BLM was shown to interact in the dHJ dissolvasome through topoisomerase 3α (TOP3α) and two OB-fold containing structural proteins, RMI1 and RMI2. In *Saccharomyces cerevisiae*, the RTR complex consists of the RecQ helicase Sgs1, Top3, and Rmi1. Several mutations of the RecQ helicase gene *BLM* cause Bloom syndrome (BS), which is a hereditary disease and associated with a predisposition for cancer (Ellis et al., 1995). A characteristic phenotype of BS cells is the elevated frequency of sister chromatid exchanges (SCEs; Chaganti et al., 1974), which result from COs between the chromatid arms. In yeast, the loss of Sgs1 also leads to an elevated recombination frequency and hypersensitivity against genotoxic agents (Onoda et al., 2000). Beside the catalytic activities required for the branch migration of the HJs, the RecQ helicase also plays a crucial role in mediating protein-protein interactions inside the RTR complex.

The third conserved complex partner of the RTR complex RMI1, which is required for the stabilization of the complex, possesses no catalytic function itself, but dHJ dissolution is absolutely dependent on it (Chang et al., 2005; Mullen et al., 2005; Yin et al., 2005; Raynard et al., 2006; Wu et al., 2006; Bussen et al., 2007; Chen and Brill, 2007). Comparable to mutants of the respective RecQ helicase involved in the RTR complex, the loss of RMI1 also leads to an elevated frequency of recombination events (Chang et al., 2005; Yin et al., 2005). RMI2 was identified as a fourth RTR complex partner in mammals and is also required for the assembly of the RTR complex and the stimulation of dissolution (Singh et al., 2008; Xu et al., 2008). The RTR complex is additionally involved in further, early steps of recombination, such as end resection, which could be shown in yeast (Zhu et al., 2008). In this process, the catalytic activity of Sgs1, stimulated by Top3 and Rmi1, is required to generate a substrate for the nucleolytic degradation of the 5′ strand by the endonuclease Dna2 (Cejka et al., 2010; Niu et al., 2010).

## THE ROLE OF THE RTR COMPLEX IN SOMATIC PLANT CELLS

The plant RecQ helicase RECQ4A from *Arabidopsis thaliana* could be identified as a functional counterpart of BLM and Sgs1 and was shown to be required for RTR functions in somatic cells (Hartung et al., 2007a; Knoll and Puchta, 2011). The loss of AtRECQ4A leads to a hypersensitivity against DNA damaging agents and to an elevated frequency of HR events (Bagherieh-Najjar et al., 2005; Hartung et al., 2007a). In rice, mutants of the RecQ homolog *OsRECQL4* also display defects after treatment with genotoxins and in suppression of HR. Furthermore, *Osrecql4* mutants exhibit an increased appearance of unrepaired double strand breaks (DSBs) and cell death (Kwon et al., 2013). In different organisms, the loss of both the endonuclease MUS81 and the RecQ helicase involved in the RTR complex leads to lethality. This synthetic lethality was also shown in *Arabidopsis* (Hartung et al., 2006). The lethality of the *mus81 recq4A* double mutant can be rescued by an additional mutation of the RAD51 paralog RAD51C (Mannuss et al., 2010), which is involved in early steps of HR. This supports the hypothesis that RECQ4A and MUS81 act in independent pathways to process toxic DNA intermediates which arise during HR.

In yeast, mutants of the RTR complex partner *Top3* exhibit a slow growth phenotype, which can be suppressed by an additional mutation of the interacting RecQ helicase, Sgs1 (Gangloff et al., 1994). A similar genetic interaction could be confirmed in plants, as the lethal phenotype of the *Arabidopsis* mutant *top3A* can be rescued by a *RECQ4A* mutation (Hartung et al., 2007a). The characteristic phenotype of the *top3/top3A* single mutant was explained by the catalytic action of the RecQ helicase, which irreversibly generates a toxic DNA intermediate that remains unresolved in the absence of the topoisomerase.

The function of the plant RecQ helicase in HR seems to be versatile. It was shown that RECQ4A is required for the efficient processing of the synthesis-dependent strand-annealing (SDSA) pathway of HR (Mannuss et al., 2010). Furthermore, the rice homolog seems to be involved in the initial step of HR, as the overexpression of *OsRECQL4* can enhance the DSB processing function, probably by promoting 5′ resection (Kwon et al., 2012). RECQ4A seems to possess at least two different and independent sub-functions required for the suppression of HR, which are dependent on the N-terminal region and the helicase activity of RECQ4A, respectively. It could also be shown *in vitro* that RECQ4A has an ATP-dependent helicase activity and it is able to catalyze fork regression (Schröpfer et al., 2013).

*Arabidopsis thaliana *contains a paralog of RECQ4A named RECQ4B. Despite a high sequence similarity and conserved domain structure of the two proteins, mutants of *RECQ4B* display no somatic DNA repair deficiency, have a somatic HR frequency lower than WT and no meiotic defects (Hartung et al., 2007a).

The two protein partners of RECQ4A in the RTR complex, TOP3α and RMI1, share many somatic functions with the helicase. Like in *recq4A* plants, in *top3A* and *rmi1* mutants, elevated sensitivity against treatment with the methylating agent methyl methanesulfonate (MMS) and the DNA crosslinking agent cisplatin can be found. In addition, loss of TOP3α or RMI1 leads to an increase in the spontaneous HR frequency to a similar level of *recq4A* plants. In addition, *top3A-2* mutants show dwarfing, curled leaves, fasciated organs, and are sterile. Investigating cell division in this mutant line showed a strong increase in the number of mitotic anaphase defects (Hartung et al., 2008).

Investigations on the role of the protein domains identified in RMI1 showed a surprising diversity of functions. Three different domains could be identified in animal and plant RMI1 homologs to date. The first OB-fold domain (OB1) is necessary in animals for the interaction of RMI1 with the RecQ helicase and topoisomerase (Yin et al., 2005; Raynard et al., 2008). N-terminal to the OB1 domain is a domain of unknown function 1767 (DUF1767), which can be found in a number of proteins N-terminal of proposed nucleic acid binding domains such as the OB-fold domain (Bonnet et al., 2013). At the RMI1 C-terminus a second OB-fold domain (OB2) is localized which has been shown to be necessary for the interaction of animal RMI1 and RMI2 (Xu et al., 2008). Interestingly, both the DUF1767 and the OB1 domain are essential for the DNA repair functions of RMI1, while there is only a weak requirement for the OB2 domain in DNA repair. Furthermore, the deletion of the OB2 domain does not affect RMI functions in somatic HR, whereas the DUF1767 and OB1 domains are both partially and non-redundantly required for the HR suppression function of RMI1 (Bonnet et al., 2013).

## THE UNIQUE ROLE OF RMI1 AND TOP3α IN PLANT MEIOSIS

In addition to the functions of the *Arabidopsis* RTR complex in somatic cells, two of its components – TOP3α and RMI1 (hereafter abbreviated as TR proteins) – are also essential for meiotic recombination. Of the two *top3A* mutant lines described so far, the hypomorphic *top3A-2* mutant produces sterile flowers in addition to the above described somatic defects (Hartung et al., 2008). In two independent studies, also mutants of *Arabidopsis* *RMI1* were shown to be defective in meiosis (Chelysheva et al., 2008; Hartung et al., 2008).

Investigation of meiosis progression of TR mutants showed remarkable defects: following wild type-like prophase I with formation of synapsis between homologous chromosomes and arrangement of bivalents at the cell equator at metaphase I, the loss of TR proteins becomes visible at anaphase I. While in wild type cells bivalents are separated and the homologous chromosomes are pulled toward opposite cell poles, in TR mutant cells the separated chromosomes stay connected by chromatin bridges and a large amount of broken chromatin fragments are found near the equator (Chelysheva et al., 2008; Hartung et al., 2008). Additional mutation of earlier meiotic genes *SPO11-1*, *SPO11-2*, and *RAD51* (Grelon et al., 2001; Li et al., 2004; Stacey et al., 2006; Hartung et al., 2007b) in *top3A* and *rmi1* mutants, respectively, showed the dependence of TR mutant phenotypes on meiotic recombination initiation. The DMC1 recombinase is a RAD51 paralog specific for meiotic recombination. *Arabidopsis* *dmc1* mutants differ from *rad51* mutants, though. Instead of severe chromosome fragmentation visible in *rad51* plants, *dmc1* mutants have univalent chromosomes because they are not able to initiate HR between homologous chromosomes, but the already formed DSBs are repaired using the sister chromatid in a RAD51-dependent manner (Couteau et al., 1999). Interestingly, while *RAD51* mutation could rescue the *rmi1* phenotype, this was not possible by a *DMC1* mutation. The *rmi1 dmc1* double mutant has univalent chromosomes that still show chromatin bridges and chromosome fragmentation in anaphase I. This is a hint that RMI1 (and presumably TOP3α) function might be required in recombination reactions involving both the homologous chromosome as well as the sister chromatid.

Since meiotic recombination is tightly coupled with synapsis of the homologous chromosomes in meiotic prophase I, the effect of a *RMI1* mutation on synapsis was analyzed. Investigation of the axial element of the synaptonemal complex protein ASY1 as well as the central element protein ZYP1 by immunolocalization revealed no changes in synapsis in *rmi1* meiocytes (Chelysheva et al., 2008). This again indicates that meiotic recombination is initiated normally when TR proteins are missing.

Following such severe damage at anaphase I, *top3A*, and *rmi1* cells finish meiosis I through the formation of dyads, but they never enter meiosis II. No cell in stage of meiosis II was ever found in *top3A* or *rmi1* mutants (Chelysheva et al., 2008; Hartung et al., 2008). Such a phenotype was very unexpected, since only very few *Arabidopsis* mutants with meiotic phenotypes have been described that do not finish meiosis irrespective of the damage to their DNA. It is therefore tempting to speculate if the arrest in TR mutants might involve a cell cycle checkpoint mechanism, especially since another mutant showing a meiosis I arrest is a cyclin A1;2 mutant, *tam* (Bulankova et al., 2010; d’Erfurth et al., 2010).

Similar to studies on the role of specific domains in RMI1 in DNA repair and HR in somatic cells, the functions of RMI1 domains was also analyzed in meiosis. While *rmi1-1* mutant plants are sterile, expression of wild type *RMI1* in this mutant background could restore fertility to wild type levels. On the other hand, plants expressing a *RMI1* construct missing the DUF1767, the OB1 or both domains remained infertile. Interestingly, plants expressing a *RMI1* construct without the OB2 domain, which in animals is necessary for the interaction with RMI2, were as fertile as wild type. Similar to these observations, analysis of prepared meiocytes from these different lines revealed that plants expressing the wild type RMI1 or the RMI1ΔOB2 construct in a *rmi1* background had a normal progression through meiosis I without chromosome fragmentation and also finished meiosis II normally, while plants missing the DUF1767, the OB1 or both domains showed meiotic defects similar to the *rmi1* mutant (Bonnet et al., 2013).

In light of previous studies from other eukaryotes, the severe meiotic phenotype of the**TR mutants in *Arabidopsis* was extremely surprising. As these kinds of mutations have not been studied in other plants yet, it is not clear how conserved the phenotype is, but it is tempting to speculate that this is a general property at least of vascular plants. On the other side, although multiple investigations of the RTR complex have been performed in various eukaryotes, a similar phenotype was not described in literature for any other organism yet. This indicates that the process is either specific for plants or has at least a much more prominent role there than in other eukaryotes.

## WHAT KINDS OF INTERMEDIATES ARE DISSOLVED IN MEIOSIS?

Double mutant analysis of TR mutants with other meiotic mutants indicates that the defect results from imperfect dissolution of recombination intermediates (see above). Taking the classical role of the RTR complex into account, the dissolved intermediates might be dHJs. However, as the *recq4A* mutant of *Arabidopsis* has only a mild telomeric meiotic defect (Higgins et al., 2011), a phenotype which is not related to the two other TR mutants, the classical RTR complex of *Arabidopsis* does not seem to be involved in the reaction. So either there is one or several alternative helicases that are able to transform a dHJ intermediate into a hemicatenane in place of RECQ4A, or for the processing of the intermediates no helicase is required at all. It is possible, for example, that mechanical forces operating during metaphase I and anaphase I to pull homologous chromosomes apart, are sufficient to facilitate branch migration of HJs without the action of a DNA helicase. This would indirectly lead to the formation of hemicatenane structure which can be processed by the TR proteins. Alternatively, the classical dHJ structure, which is also an intermediate of CO resolution in meiosis, might not be the structure the TR proteins are working on in *Arabidopsis*. Recent reports indicate that hemicatenanes can be dissolved without assistance of a helicase by type IA DNA topoisomerases (Glineburg et al., 2013; Lee et al., 2013). So the most probable explanation is that the meiotic intermediate processed by the TR proteins is still a hemicatenane, but it might not be formed by branch migration of dHJs. In support of this idea it was shown that although *rmi1* mutants form bivalents connecting the homologous chromosomes in meiosis I, these bivalents are also formed in the absence of ZMM pathway proteins MSH5 and MER3 that are thought to be required for the formation of COs *via* dHJs (Chelysheva et al., 2008). Therefore, bivalents formed in *rmi1* plants might not connect homologous chromosomes by dHJs at all, but by some other form of complex joint molecules. Since the meiotic TR mutant phenotype is dependent on meiotic recombination initiation, it is improbable that hemicatenanes are processed that were generated at replication forks in premeiotic S-phase.

## THE NATURE OF GENE CONVERSIONS – ANOTHER SURPRISING PECULIARITY OF PLANT MEIOTIC RECOMBINATION

Meiotic recombination is induced by DSBs and the resulting intermediates between homologous chromosomes can be resolved into either COs or NCOs. By applying complete genome sequencing techniques, the number of COs and NCOs during a single meiosis can be determined. Whereas in *S. cerevisiae* there is a relation of about one CO to NCO, in mammals the number of NCOs outweighs the number of COs [reviewed in de Massy (2013)]. In both organisms these numbers can be set into relation with the total number of DSBs induced per meiosis. Interestingly, most studies performed in *Arabidopsis* reveal a different picture. It is generally accepted that about 150 to 200 DSBs occur during meiosis and about 10 COs arise (Chelysheva et al., 2007; Sanchez-Moran et al., 2007; Vignard et al., 2007). It was therefore obvious to postulate that the repair of most DSBs results in NCOs. Surprisingly, in several independent studies using single nucleotide polymorphisms (SNPs) between different *Arabidopsis* cultivars as markers, sequence analysis of meiotic recombinants revealed hardly any NCO events whereas the number of COs were in the expected range (Lu et al., 2012; Salome et al., 2012; Choi et al., 2013; Drouaud et al., 2013; Wijnker et al., 2013). If NCO GC tracts would have sizes in the range of NCO events in either yeast or mammals, the studies should have revealed NCO numbers in the range of DSBs induced in meiosis. As this was not the case, there are two possible scenarios to explain the data: either the mean conversion tract length in *Arabidopsis* is so short that using SNPs for detection, most events have been missed, or that most DSBs are not repaired using the homologous chromosome as recombination partner, but the sister chromatid. Indeed recombination between sister chromatids has been documented in meiosis in yeast (Goldfarb and Lichten, 2010) and *Arabidopsis* (Cifuentes et al., 2013).

## TWO SPECIFIC PECULIARITIES – TWO SIDES OF THE SAME COIN?

It is tempting to speculate that the NCO peculiarities in *Arabidopsis* are somehow linked to the presence of a unique TR dissolution pathway in meiotic recombination. Due to the severe phenotype of the mutants, TR-mediated dissolution seems to be required for the processing of intermediates resulting from large portions or even the majority of the DSBs induced in meiosis. In principle, such a kind of intermediate could result from recombination between sister chromatids. As in somatic cells the functional RTR complex is involved in suppression of SCEs in various organisms, a related mechanism might operate as backup repair mechanism during meiotic recombination in plants. Alternatively, RTR complex-mediated activity might produce recombination products of homologous chromosomes where the tract length of the information transferred from one parental chromosome to the other is quite short. Longer tracts would then be observed if dHJ are resolved by an endonuclease or if a NCO is produced by a SDSA mechanism. Thus, the reason why most GCs are not detectable with the classical SNP technology applied in current studies in *Arabidopsis* might be the meiosis-specific activity of the TR proteins.

## Conflict of Interest Statement

The authors declare that the research was conducted in the absence of any commercial or financial relationships that could be construed as a potential conflict of interest.
